# Characterization of relaxin 3 and its receptors in chicken: Evidence for relaxin 3 acting as a novel pituitary hormone

**DOI:** 10.3389/fphys.2022.1010851

**Published:** 2022-11-07

**Authors:** Can Lv, Huilu Zheng, Biying Jiang, Qin Ren, Jiannan Zhang, Xin Zhang, Juan Li, Yajun Wang

**Affiliations:** ^1^ Key Laboratory of Bio-Resources and Eco-Environment of Ministry of Education, College of Life Sciences, Sichuan University, Chengdu, China; ^2^ Animal Disease Prevention and Food Safety Key Laboratory of Sichuan Province, College of Life Sciences, Sichuan University, Chengdu, China; ^3^ Joint Nutrition Center for Animal Feeding of Sichuan University-Shengliyuan Group, Chengdu, China

**Keywords:** chickens, RLN3, RXFP1, RXFP3, pituitary

## Abstract

Mammalian relaxin (RLN) family peptides binding their receptors (RXFPs) play a variety of roles in many physiological processes, such as reproduction, stress, appetite regulation, and energy balance. In birds, although two relaxin family peptides (RLN3 and INSL5) and four receptors (RXFP1, RXFP2, RXFP2-like, and RXFP3) were predicated, their sequence features, signal properties, tissue distribution, and physiological functions remain largely unknown. In this study, using chickens as the experimental model, we cloned the cDNA of the *cRLN3* gene and two receptor (*cRXFP1* and *cRXFP3*) genes. Using cell-based luciferase reporter assays, we demonstrate that cRLN3 is able to activate both cRXFP1 and cRXFP3 for downstream signaling. cRXFP1, rather than cRXFP3, is a cognate receptor for cRLN3, which is different from the mammals. Tissue distribution analyses reveal that *cRLN3* is highly expressed in the pituitary with lower abundance in the hypothalamus and ovary of female chicken, together with the detection that cRLN3 co-localizes with pituitary hormone genes *LHB/FSHB/GRP/CART* and its expression is tightly regulated by hypothalamic factors (GnRH and CRH) and sex steroid hormone (E2). The present study supports that cRLN3 may function as a novel pituitary hormone involving female reproduction.

## Introduction

Relaxin (RLN) was originally named in 1930 for its role in promoting the relaxation of the pubic ligament during pregnancy in mammals (e.g., guinea pigs and rabbits) (Fevold et al., 1930). Later studies report that RLN belongs to the family of peptide hormones that also include insulin and insulin-like growth factors (IGFs). The crystal structure of RLN is similar to that of insulin including the alpha helices of the A- and B-chain which are mutually supported by the two interchain cysteine bridges and one intrachain bridge in the A-chain (Haley et al., 1982; Hudson et al., 1983; Patil et al., 2017). Increasing evidence shows that RLNs play important roles in many physiological functions, such as promoting pregnancy response, metabolism, stress, and energy balance (van der Westhuizen et al., 2008; Bathgate et al., 2013; Patil et al., 2017). In vertebrates, the relaxin family peptides vary significantly among species due to multiple (2 or 3) rounds of whole genome duplication and gene gain/loss. Till present, a total of seven structurally related peptides, relaxin 1 (RLN1), RLN2, RLN3, insulin-like peptide 3 (INSL3), INSL4, INSL5, and INSL6, have been identified in humans (Patil et al., 2017). Three RLNs (RLN1, RLN2, and RLN3) exist in higher primates (e.g., great apes), while only RLN1 and RLN3 exist in some mammals such as mice and pigs (Wilkinson et al., 2005; Patil et al., 2017). As the main form in the blood circulation and corpus luteum, RLN1 and RLN2 (in higher primates) are collectively referred to as RLN among these species (Bathgate et al., 2013).

As the typical member of the insulin superfamily, the RLNs bind their receptors to play their physiological roles. In mammals, RLN1 and RLN2 bind the relaxin family peptide 1 receptor (RXFP1) for downstream signaling (Bathgate et al., 2013). As their cognate receptor, RXFP1, which is also called the leucine-rich repeat-containing G-protein-coupled receptor 7 (LGR7), is capable of coupling to multiple G-proteins, thus triggering the pathways including cAMP accumulation (Halls et al., 2006; Halls et al., 2007; Halls et al., 2009), ERK1/2 phosphorylation, NO production, and other signaling pathways (Bathgate et al., 2013). In humans, RLN3 mainly binds the relaxin family peptide 3 receptor (RXFP3) for downstream signaling, which is coupled to the Gα_i/o_ protein with cAMP level decrease upon ligand binding (Liu et al., 2003b; Liu et al., 2005; Bathgate et al., 2006; van der Westhuizen et al., 2007). In HEK293T cells, RLN3 was also able to activate RXFP1 inducing a dose-dependent increase in cAMP production (Sudo et al., 2003). Till present, INSL3 is reported to bind the relaxin family peptide 2 receptor (RXFP2) for downstream signaling including cAMP level increase and thymidine incorporation (Kumagai et al., 2002; Hsu et al., 2003). However, the cognate receptors for INSL4 and INSL6 remain to be identified (Bathgate et al., 2013).

In the 1980s, two RLN genes, *RLN1* and *RLN2*, were isolated from the human genome (Crawford et al., 1984). Later, the *RLN3* gene was further isolated (Bathgate et al., 2002). Sequence analyses reveal that the *RLN3* gene is the ancestral gene which continually duplicates, thus bringing the enrichment of RLN family peptides in mammals (Wilkinson et al., 2005). In humans, RLN3 is mainly localized within the brain and is involved in physiological roles including stress, motivated behavior, and appetite regulation (Smith et al., 2011; Ryan et al., 2013; McGowan et al., 2014; Smith et al., 2014; Calvez et al., 2016). As the ancestral gene copy, the *RLN3* gene also duplicates in many lower vertebrates including fish (Wilkinson et al., 2005). For example, in teleost fish, two peptides closely related to relaxin 3, RLN3a and RLN3b, have been identified (Yang et al., 2020).

In chickens, a relaxin-like peptide has been partially purified from the ovaries of actively laying hens by size-exclusion chromatography (Brackett et al., 1997). In addition, RLN3 is reported to be related to the transformation of brooding and laying eggs (Shen et al., 2016; Ye et al., 2019). In contrast to the detailed and extensive investigation of the RLN3 peptide in mammals, the sequence features, signaling properties, and expression profiles of R*LN3* within the avian species remain poorly understood. Therefore, using chicken as an experimental model, our present study aims to 1) identify the complete ORF sequences of *RLN3* and its putative receptors: *RXFP1* and *RXFP3*; 2) reveal the signaling properties of RLN3; and 3) explore the tissue distribution and regulation mechanism of *RLN3* expression. The results from our study are the first to establish a clear concept that avian RLN3 is a novel pituitary hormone which may be involved in female reproduction.

## Materials and methods

### Ethics statement

All the animal experiments were conducted in accordance with the Guidelines for Experimental Animals issued by the Ministry of Science and Technology of the People’s Republic of China. The experimental protocol was approved by the Animal Ethics Committee of Sichuan University (Chengdu, China).

### Chemicals, peptides, and primers

All chemicals were purchased from Sigma-Aldrich (St. Louis, MO), and restriction enzymes were purchased from Takara Biotechnology Co., Ltd., (Dalian, China). The recombinant human relaxin 3 (rhRLN3) was purchased from Roche R&D Center (China) Ltd. Chicken gonadotropin-releasing hormone (GnRH, GnRH1 was used in this study) and corticotropin-releasing hormone (CRH) were synthesized using solid-phase Fmoc chemistry (GL Biochem, Shanghai, China). 17β-estradiol (E2) and progesterone (P4) were purchased from Sigma-Aldrich. dihydrotestosterone (DHT) was purchased from Cayman Chemical Company (Ann Arbor, Michigan, United States). In this study, all chickens of the Lohmann Layer strain were purchased from a local commercial company (Chengdu, China). All primers used in this study were synthesized by Youkang Biological Technology Co., Ltd., (Hangzhou, China) and are listed in Table 1.

**TABLE 1:** Primers used in this study.

Gene name	Sense/antisense	Primer sequence (5′-3′)	Size (bp)
Primers for construction of the expression plasmids			
*cRLN3*	Sense	CCG​GAA​TTC​AAC​GGA​ACG​GCG​ACG​CGC​CAT	621
	Antisense	CCG​GAA​TTC​CAT​GCA​CAA​GTG​CAG​AGA​GG	
*cRXFP1*	Sense	CCG​GAA​TTC​GCT​GAA​AGA​CAG​ATA​TGA​CAT​C	2,317
	Antisense	CCG​GAA​TTC​CCC​AAA​CAT​ATT​TAC​GTG​TAC​G	
*cRXFP3*	Sense	CCG​GAA​TTC​AGC​ATG​GGA​TGG​ATG​AGC​TC	1,341
	Antisense	CCG​GAA​TTC​GCC​ATC​TCA​GTA​GTG​TTG​CT	
Primers for quantitative real-time RT-PCR assay			
*cRLN3*	Sense	ATT​CTT​CTC​AAG​CAG​CAA​GT	169
	Antisense	TCT​TTG​AAG​TCA​TCT​GCC​AT	
*cRXFP1*	Sense	GCT​CCA​CGC​CAT​CTC​AAT​AA	160
	Antisense	CAG​CGA​TCC​CAC​CAA​TTG​AC	
*cRXFP3*	Sense	GTT​GGC​AAT​CGT​GGC​TTC​TC	135
	Antisense	GTT​TGG​TAC​AGA​CCC​AGC​CA	
*cβ-actin*	Sense	CCC​AGA​CAT​CAG​GGT​GTG​ATG	123
	Antisense	GTT​GGT​GAC​AAT​ACC​GTG​TTC​AAT	

### Total RNA extraction

In order to explore the expression of *cRLN3* and its receptors in various tissues of chickens, three adult male and three adult female chickens were purchased from local companies. All chickens were sacrificed and the tissues were isolated, including the various brain regions (telencephalon, midbrain, cerebellum, hindbrain, and hypothalamus), pituitary, heart, liver, spleen, lung, kidney, skin, muscle, adrenal gland, pancreas, duodenum, abdominal fat, spinal cord, testis, ovary, infundibulum, isthmus, uterus, and vagina. All tissue samples were stored at −80°C before use. The total RNA was extracted with RNAzol reagent (Molecular Research Center, Cincinnati, OH) according to the manufacturer’s instructions and diluted in H_2_O treated with diethylpyrocarbonate (DEPC). Briefly, after precipitating with DEPC-treated ultra-pure water, the RNAzol-lysed tissues were centrifuged (12,000 rpm, 10 min). BAN solution (4-bromoanisole) was added to purify the RNA and eliminate genomic DNA. Then, an equal volume of cold 100% isopropanol was added. The precipitated pellet was washed three times with 600 μl 75% ethanol and dissolved in 30 μl RNase-free water. The approximate concentration and purity of samples were determined using a OneDrop 1000 spectrophotometer.

### Reverse transcription and quantitative real-time PCR

For reverse transcription, total RNA (2 μg) and 0.5 μg of oligo-deoxythymidine were mixed in a volume of 5 μl, incubated at 70°C for 10 min, and cooled at 4°C for 2 min. Then, the first strand buffer, 0.5 mM each deoxynucleotide triphosphate (dNTP), and 100 U Moloney murine leukemia virus (MMLV) reverse transcriptase (Takara) were added to the reaction mix in a total volume of 10 μl. Reverse transcription (RT) was performed at 42°C for 90 min. After the reaction, the cDNA templates were diluted with MilliQ-H_2_O and stored at −20°C.

According to our previously established method (Liu et al., 2019), quantitative real-time PCR was performed to examine the expression of *RLN3, RXFP1*, and *RXFP3* mRNA among chicken tissues. The qPCR primers of chicken *RLN3*, *RXFP1*, and *RXFP3* were designed based on sequences in the GenBank and are listed in Table 1. The primers (10 μM), dNTP (10 mM), easy Taq buffer, easy Taq DNA polymerase (TransGen Biotech), Eva Green (Biotium), MilliQ-H_2_O, and templates were mixed in a total volume of 20 μl. Then the reaction mix was conducted on the CFX96 Real-Time PCR Detection System (Bio-Rad). The amplification conditions included an initial denaturation for 10 min at 94°C followed by 20 s of denaturation at 94°C, 15 s annealing at 60°C, and 30 s extension at 72°C for 40 cycles.

### Cloning the cDNA of chicken *RLN3* and its receptors (*RXFP1* and *RXFP3*)

According to the GenBank database, a relaxin peptide (RLN3: NM_001113200.1) and its two putative receptors (RXFP1: XM_420385.5; RXFP3: XM_004937174.3) exist in chickens. Based on the predicted cDNAs of these genes, gene-specific primers were designed to amplify the CDS of *cRLN3*, *cRXFP1*, and *cRXFP3* with high-fidelity DNA Taq polymerase (TOYOBO, Japan). Then, these amplified PCR products were inserted into the pcDNA3.1 (+) expression vector (for functional assays) and sequenced by Youkang Biological Technology Co., Ltd. Finally, the CDS region of each gene was determined by sequencing at least three independent clones.

### Preparation of chicken RLN3 conditioned medium

To verify whether chicken RLN3 is functional, the conditioned medium of cells expressing cRLN3 (cRLN3_cm_) was prepared. In brief, the expression plasmid of cRLN3 (1,000 ng) was transfected into HEK293 cells cultured in a 6-well plate. After 6 h transfection, the culture medium with 15% fetal bovine serum was replaced and continued for 18 h at 37°C with 5% CO_2_. Then, the serum-rich medium was replaced with 600 μl of serum-free medium. After 24 h culture, the conditioned medium containing cRLN3 was collected and concentrated by centrifugation in an Amicon Ultra 3K (Millipore, United States) ultrafiltration tube at 4°C, 5,000 g for 30 min, with a final volume of approximately 1/4 of the original. The concentrated medium was used for subsequent functional tests. Using the same approach, the conditioned medium of cells transfected with empty pcDNA3.1 (+) expression plasmid (pcDNA_cm_) was used as a negative control.

### Functional characterization of chicken RLN3 and its receptors (RXFP1 and RXFP3)

According to our previously established methods (Liu et al., 2019; Jiang et al., 2022), two cell-based luciferase reporter systems (pGL3-CRE-luciferase and pGL3-NFAT-RE-luciferase) were used to verify whether cRLN3_cm_ is biologically active and capable of activating cRXFP1 and cRXFP3. In brief, HEK293 cells transiently expressing each receptor (cRXFP1 and cRXFP3) were treated with 30 μl serum-free medium containing cRLN3_cm_ or pcDNA_cm_ (0.001–10 μl) for 6 h, and the receptor-activated cAMP/PKA pathways and calcium mobilization were then monitored by pGL3-CRE-luciferase and pGL3-NFAT-RE-luciferase reporter systems, respectively. HEK293 cells expressing empty pcDNA3.1 (+) were used as a negative control.

To compare the functional difference between chicken RLN3 and human RLN3, recombinant human RLN3 (rhRLN3, 10^–12^ to 10^–7^ M, 6 h) was used to treat HEK293 cells expressing cRXFP1 or cRXFP3, respectively, and its potency in activating cRXFP1 and cRXFP3 was also evaluated.

### Preparation of the extracellular medium from chicken pituitary cells

To explore whether pituitary cells can secrete RLN3, we collected the extracellular medium of adult female chicken pituitary cells to detect its activation potential. According to the method provided in the previous article (Sun et al., 2021; Liu et al., 2022), the anterior pituitaries from a laying hen were isolated and further cut into small pieces with clean scissors. After washing with PBS, the cut pieces were then digested with 0.25% trypsin at 37°C for 30 min. The dispersed pituitary cells were cultured in Medium 199 supplemented with 10% fetal bovine serum in a Corning Cell BIND 48-well plate (Corning, Tewksbury, MA) at 37°C with 5% CO_2_. After 18-h culture, the medium was replaced with a serum-free M199 medium. Four hours later, this extracellular medium from chicken pituitary cells was collected and loaded into Amicon Ultra 3K (Millipore, United States) ultrafiltration tubes for concentration by centrifugation at 4°C, 5,000 g, for 15 min, to a final volume of approximately 1/2 of the original. The HEK293 cells expressing cRXFP1 were treated with a 60 μl serum-free medium containing chicken pituitary cell extracellular medium (2 × 10^−3^μl to 2 × 10^1^ μl) for 6 h, and then receptor-activated calcium mobilization was monitored by pGL3-NFAT-RE-luciferase reporter systems. HEK293 cells expressing empty pcDNA3.1 (+) were used as a negative control.

### Effects of hypothalamic factors (gonadotropin-releasing hormone/corticotropin-releasing hormone) and sex hormone (17β-estradiol/progesterone/dihydrotestosterone) on *cRLN3* expression in cultured chicken pituitary cells

To explore the regulatory effects of GnRH/CRH/E2/P4 on cRLN3, the anterior pituitaries isolated from 4-week-old female chicks were sliced and digested by 0.25% trypsin at 37°C for 20 min. The dispersed pituitary cells were cultured at a density of 5 × 10^5^ cells/well in Medium 199 supplemented with 15% fetal bovine serum. Then, the medium was replaced with a serum-free M199 medium and the cells were incubated with GnRH (1–100 nM), CRH (0.1–10 nM), E2 (100 nM), and P4 (100 nM) for different duration times (4 h/24 h). In the present study, the pituitary cells from 4-week-old male chicks were also treated with DHT (100 nM) for 4 h/24 h. The total RNA was then extracted from cultured pituitary cells and the expression of *RLN3* and β-actin mRNA was assayed by qPCR.

### Data analysis

The luciferase activities of HEK293 cells expressing cRXFP1/cRXFP3/pcDNA3.1 (+) in the treated group were expressed as a relative fold increase compared to the control group (without peptide treatment). The relative mRNA levels of target genes were first calculated as the ratios to that of β-actin and then expressed as fold change (or the percentage) compared to their respective controls (chosen tissues). The data were analyzed by Student’s t-test (between two groups), or by one-way ANOVA followed by the Dunnett test in GraphPad Prism 7 (GraphPad Software, San Diego, CA). To validate our results, all experiments were repeated twice or thrice.

## Results

### Cloning of *cRLN3*, *cRXFP1* and *cRXFP3*

**FIGURE 1:**
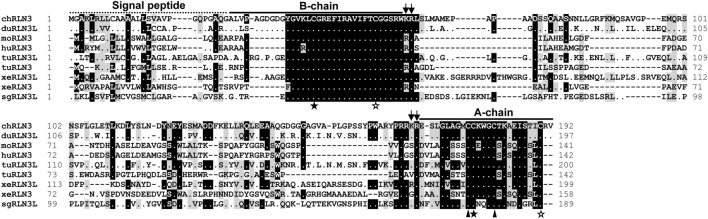
Amino acid alignment of chicken RLN3 (chRLN3) with that of ducks (duRLN3L, XP_032061192.1), mice (moRLN3, NP_775276.1), humans (huRLN3, NP_543140.1), Western painted turtles (tuRLN3L, XP_005296973.1; tuRLN3, XP_005310753.1), *Xenopus tropicalis* (xeRLN3L, NP_001072488.1; xeRLN3, NP_001116073.1), and spotted gars (sgRLN3L, XP_015192667.1). The conserved dibasic residues (KR) for proteolytic processing are marked by two arrows. The cysteine residues of two pairs of disulfide bonds for A-chain and B-chain formation are represented by solid stars and hollow stars, respectively. The two cysteine residues used to form the disulfide bond in the A-chain are represented by solid triangles. Dashes denote gaps in the alignment.

In this study, the complete ORFs of chicken (c) *RLN3*, *RXFP1*, and *RXFP3* were cloned for the first time from the chicken brain. The cloned *cRLN3* cDNA is 579 bp in length, which encodes a precursor of 192 amino acids (a.a.), with a signal peptide of 22 a.a. located at the N-terminus, followed by the B-chain, C-chain, and A-chain at the C-terminus (Figure 1). Sequence analyses demonstrated that the chicken RLN3 precursor showed a very low sequence identity (28%∼41%) with that of humans, mice, *Xenopus tropicalis*, and spotted gars. However, as shown in Figure 1, the a.a. sequence identity of the mature peptides among the species is very high. For example, the A- and B-chain of chicken RLN3 share 54% and 67% a.a. sequence identity with those of human RLN3, respectively (Table 2). Like human relaxin family peptides, the cRLN3 precursor showed the conserved two pairs of dibasic residues (K61R62 and K166R167) that were essential for proteolytic processing (Bathgate et al., 2002). In addition, similar to the insulin superfamily members, the six cysteine residues in B- and A-chains critical for the formation of the disulfide bonds (Cys43–Cys177; Cys176–Cys181; and Cys55–Cys190) were detected to be conserved among the species. Moreover, a conserved RxxxRxxI/V motif was detected in the B-chain of cRLN3.

**TABLE 2:** Amino acid sequence identity of chicken (c) RLN3 A-chain and B-chain with that of human (h) RLN1/2/3.

	hRLN1 A/B (%)	hRLN2 A/B (%)	hRLN3 A/B (%)
cRLN3 A-chain	41.18	38.24	66.70
cRLN3 B-chain	34.62	34.62	53.85

**FIGURE 2:**
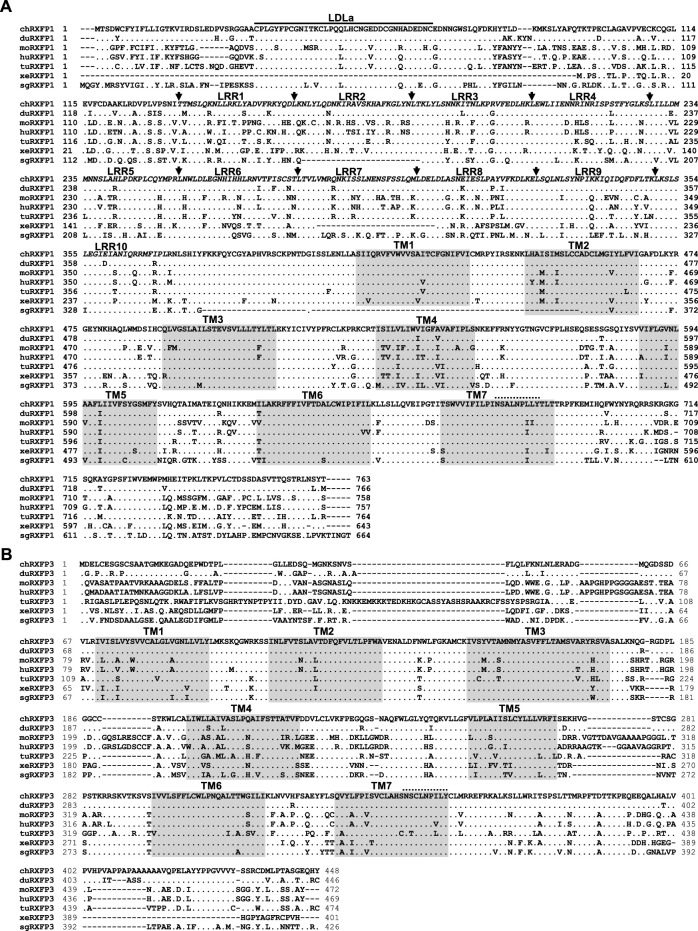
Amino acid sequence alignment of chicken RXFP1/3 with that from other species. **(A)** Amino acid alignment of chicken RXFP1 (chRXFP1) with that of ducks (duRXFP1, XP_012952098.1), mice (moRXFP1, NP_997617.1), humans (huRXFP1, NP_067647.2), Chinese soft-shell turtles (tuRXFP1, XP_006121928.1), *X. tropicalis* (xeRXFP1, XP_017951372.1), and spotted gars (sgRXFP1, XP_015200025.1). **(B)** Amino acid alignment of chicken RXFP3 (chRXFP3) with that of ducks (duRXFP3, XP_027302601.1), mice (moRXFP3, NP_848832.1), humans (huRXFP3, NP_057652.1), Chinese soft-shell turtles (tuRXFP1, XP_006116663.1), *X. tropicalis* (xeRXFP3, XP_002941625.1), and spotted gars (sgRXFP3, XP_006627308.1). The seven transmembrane domains (TM1-7) are shaded; the highly conserved NSxLNP(L/I)Y motif present in the TM7 is represented by a dashed overline; dots indicate the amino acid; dashes denote gaps in the alignment. For cRXFP1, the unique low-density lipoprotein receptor type A (LDLa) module is represented by a solid overline; the next 10 leucine-rich repeat (LRR) regions are shown in italics and separated by arrows.

In the present study, the coding region of *cRXFP1* is 2,292 bp in length encoding the receptor of 763 a.a.. Sequence analyses revealed that chicken RXFP1 shared high sequence identity with that of ducks (94%), mice (73%), humans (77%), turtles (82%), *X. tropicalis* (65%), and spotted gars (56%) (Figure 2A). As a typical G-protein-coupled receptor, cRXFP1 also showed the seven transmembrane domains (TM) and a highly conserved NSxLNP(L/I)Y motif for G-protein coupling. Like human RXFP1, chicken RXFP1 showed a very large N-terminus, including a unique low-density lipoprotein receptor type A (LDLa) module and the next 10 leucine-rich repeat (LRR) regions connected by a hinge region (Bathgate et al., 2013).

In the present study, the complete ORF of *cRXFP3* is 1,347 bp in length encoding the receptor of 448 a.a.. Sequence analyses revealed that cRXFP3 also showed high a.a. sequence identity with RXFP3 of ducks (92%), mice (59%), humans (58%), turtles (60%), *X. tropicalis* (63%), and spotted gars (66%) (Figure 2B). In the present study, as shown in Figure 2B, the conserved seven transmembrane domains (TM) and a highly conserved NSxLNP(L/I)Y motif for G-protein coupling were also detected to be conserved in cRXFP3. Unlike cRXFP1, chicken RXFP3 showed a relatively short NH_2_-terminal domain.

### Synteny analyses of *cRLN3*, *cRXFP1* and *cRXFP3*

**FIGURE 3:**
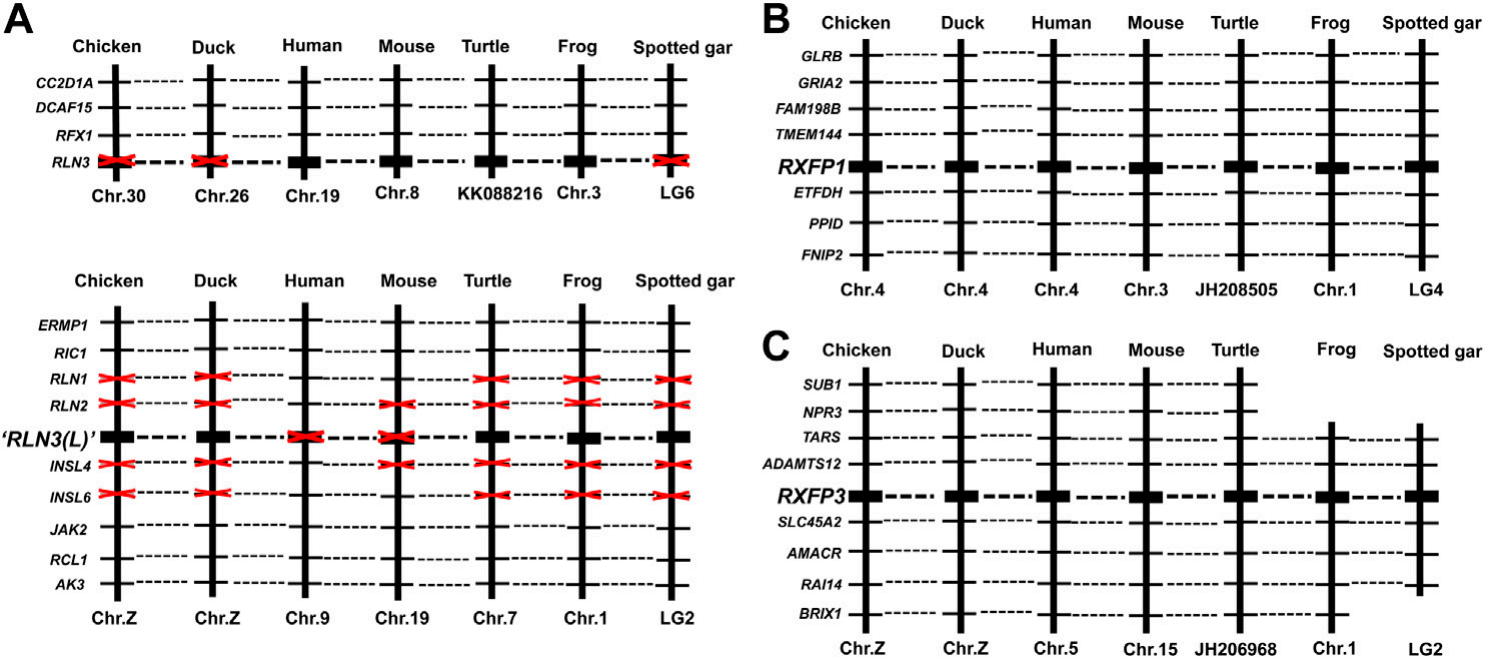
Synteny analyses of *RLN3* **(A)**, *RXFP1* **(B),** and *RXFP3* **(C)** among chicken (*Gallus gallus*), duck (*Anas platyrhynchos*), human (*Homo sapiens*), mouse (*Mus musculus*), turtle [RLN3: painted turtle (*Chrysemys picta*); RXFP1/3: Chinese soft-shell turtle (*Pelodiscus sinensis*)], frog (*Xenopus tropicalis*), and spotted gar (*Lepisosteus oculatus*).

To examine whether chicken *RLN3*, *RXFP1,* and *RXFP3* are orthologous to the genes identified in other vertebrates, synteny analyses were performed by searching their conserved neighboring genes in the genomes of human and other vertebrates, including ducks (*Anas platyrhynchos*), mice (*Mus musculus*), turtles (*Pelodiscus sinensis or Chrysemys picta*), frogs (*X. tropicalis*), and spotted gars (*Lepisosteus oculatus*) (Figure 3). Interestingly, chicken/duck *RLN3* is orthologous to *RLN3* identified in turtles, frogs, and spotted gars, but not orthologous to *RLN3* identified in humans and mice (Figure 3A). In Figures 3B,C, chicken *RXFP1* and *RXFP3* are orthologous to the genes identified in ducks, humans, mice, turtles, and frogs and spotted gars, respectively.

### Functional characterization of cRLN3, cRXFP1 and cRXFP3

To verify whether chicken RLN3 can activate RXFP1 or RXFP3, a cRLN3-conditioned medium (cRLN3_cm_, 0.001–10 μl) was used to treat HEK293 cells expressing cRXFP1/cRXFP3. Receptor activation was examined by pGL3-CRE-luciferase and pGL3-NFAT-RE-luciferase reporter systems established in our previous studies (Liu et al., 2019).

**FIGURE 4:**
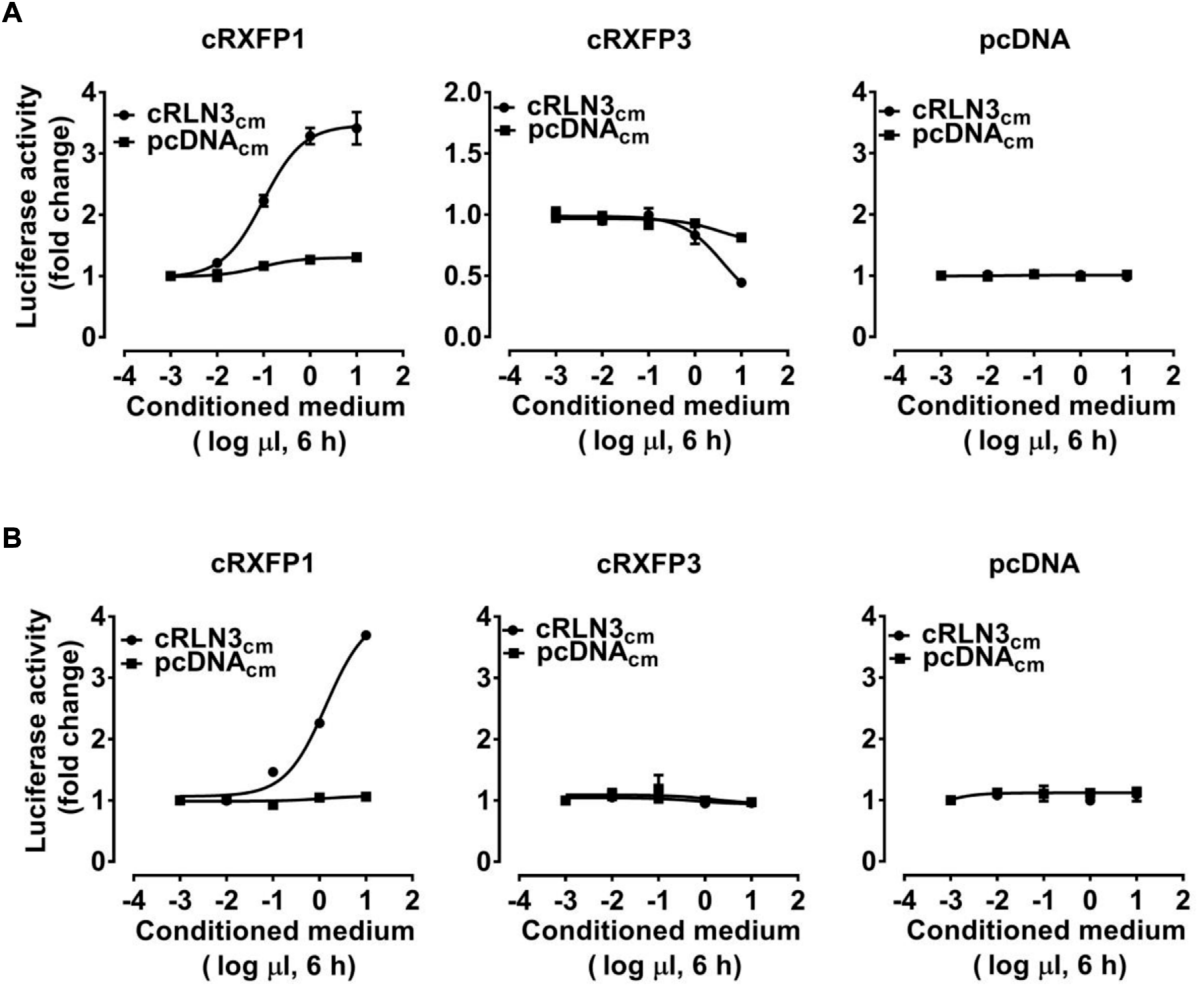
Effects of the chicken RLN3-conditioned medium (1 × 10^−3^μl to 1 × 10^1^ μl cRLN3_cm_ in 30 μl medium, 6 h) on the activation of cRXFP1, cRXFP3 expressed in HEK293 cells, monitored by a pGL3-CRE-luciferase reporter system **(A)** or a pGL3-NFAT-RE-luciferase reporter system **(B)**. HEK293 cells transfected with an empty pcDNA3.1 (+) vector were used as a control and cRLN3_cm_ treatment did not change the luciferase activity, indicating the specific action on receptor activation. Each data point represents the mean ± SEM of three replicates (*N* = 3).

As shown in Figure 4A, using a pGL3-CRE-luciferase reporter system, we demonstrated that cRLN3_cm_ could stimulate luciferase activity of HEK293 cells expressing cRXFP1 in a dose-dependent manner, while it could inhibit forskolin (2 μM, an adenlyate cyclase activator)-induced luciferase activity of HEK293 cells expressing cRXFP3 dose-dependently. The present finding indicated that cRXFP1 was coupled to the Gs-cAMP signaling pathway, while cRXFP3 was coupled to the Gi-cAMP signaling pathway. Moreover, we found that cRLN3_cm_ was at least 10-fold more potent in activating cRXFP1 than cRXFP3.

As shown in Figure 4B, using a pGL3-NFAT-RE-luciferase reporter system, cRLN3_cm_ could also stimulate the luciferase activity of HEK293 cells expressing cRXFP1 (not cRXFP3) dose-dependently, indicating that cRXFP1 activation may trigger calcium mobilization.

**FIGURE 5:**
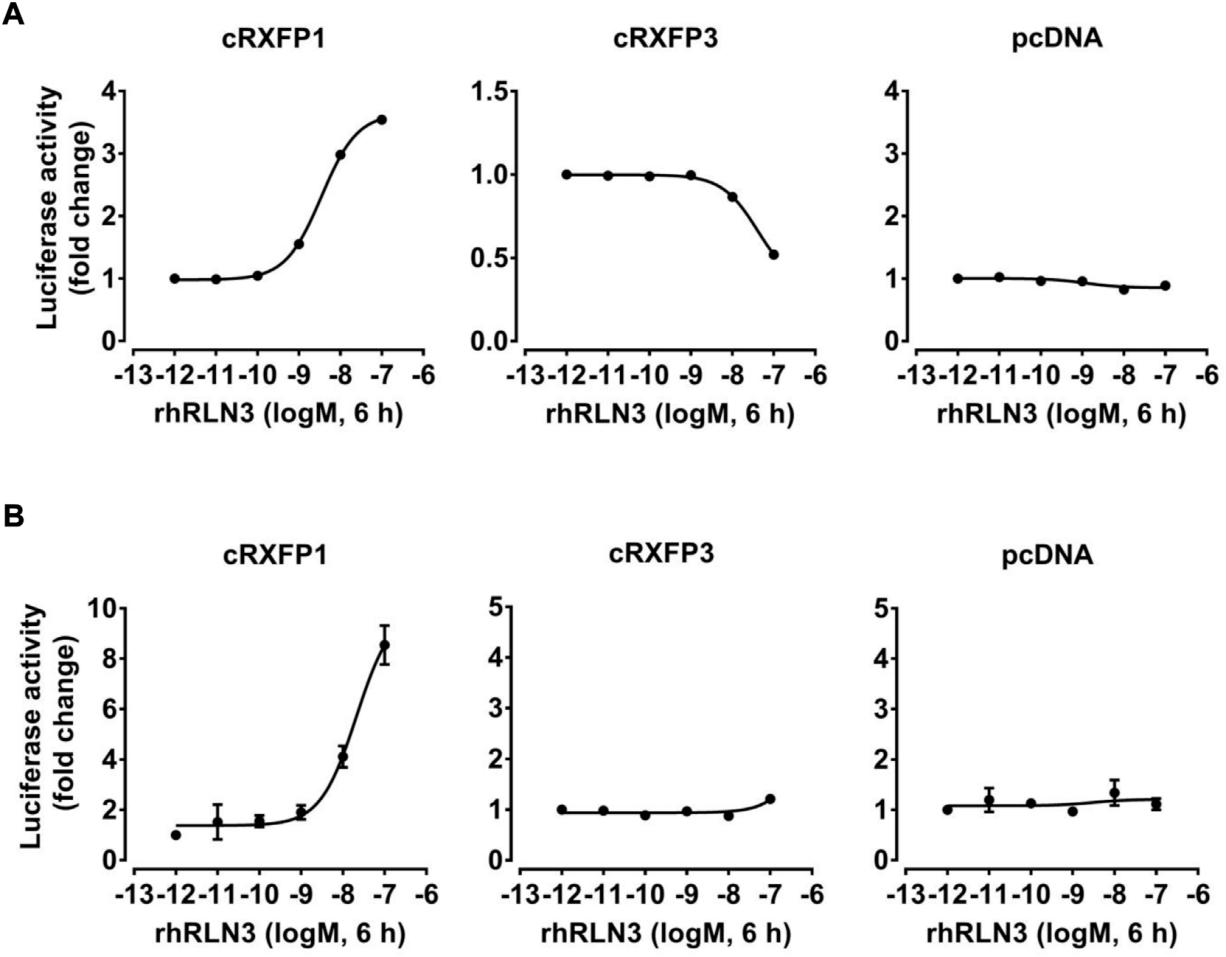
Effects of rhRLN3 (10^−12^–10^−7^ M, 6 h) on the activation of cRXFP1, cRXFP3 expressed in HEK293 cells, monitored by a pGL3-CRE-luciferase reporter system **(A)** or a pGL3-NFAT-RE-luciferase reporter system **(B)**. HEK293 cells transfected with an empty pcDNA3.1 (+) vector were used as a control and rhRLN3 treatment did not change the luciferase activity, indicating the specific action on receptor activation. Each data point represents the mean ± SEM of three replicates (*N* = 3).

Considering the close structural and evolutionary relationship between chicken RLN3 and human RLN3, we further tested whether recombinant human RLN3 (rhRLN3) could activate cRXFP1 and cRXFP3. As shown in Figure 5A, using a pGL3-CRE-luciferase reporter system, we demonstrated that rhRLN3 could potently stimulate the luciferase activity of HEK293 expressing cRXFP1 with an EC_50_ value of 2.68 nM. In addition, rhRLN3 also could inhibit forskolin-stimulated luciferase activities of HEK293 cells expressing cRXFP3 with an EC_50_ value of 41.55 nM (Table 3). Using a pGL3-NFAT-RE-luciferase reporter system, we demonstrated that rhRLN3 could stimulate luciferase activities of HEK293 cells expressing cRXFP1 (not cRXFP3) with an EC_50_ value of 21.15 nM (Figure 5B; Table 3). These findings supported that cRLN3 was functionally similar to human RLN3.

**TABLE 3:** EC_50_ values of rhRLN3 in activating different signaling pathways in HEK293 cells expressing chicken (c) RXFP1 and RXFP3.

Signaling pathway	cAMP/PKA (nM)	Ca^2+^ (nM)
cRXFP1	2.68	21.15
cRXFP3	41.55	—

### Expression of *cRLN3*, *cRXFP1* and *cRXFP3* in chicken tissues

**FIGURE 6:**
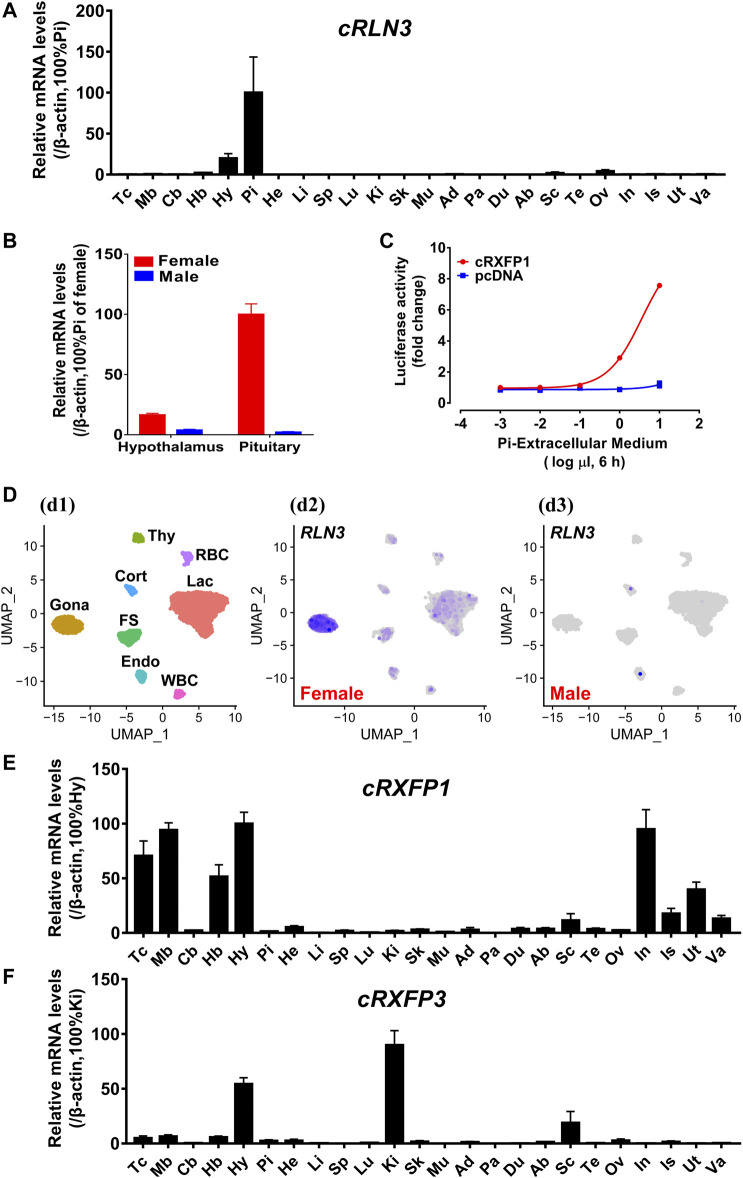
**(A)** Quantitative real-time RT-PCR (qPCR) analysis showing the abundant expression of *cRLN3* in various adult chicken tissues. Each data point represents the mean ± SEM of six individual adult chickens (three males and three females) (*N* = 6). Tc, Telencephalon; Mb, Midbrain; Cb, Cerebellum; Hb, Hindbrain; Hy, Hypothalamus; Pi, Pituitary; He, Heart; Li, Liver; Sp, Spleen; Lu, Lung; Ki, Kidney; Sk, Skin; Mu, Muscle; Ad, Adrenal gland; Pa, Pancreas; Du, Duodenum; Ab, Abdominal fat; Sc, Spinal cord; Te: Testis; Ov, Ovary; In, Infundibulum; Is, Isthmus; Ut, Uterus; Va, Vagina. **(B)** Quantitative real-time RT-PCR analysis showing the sex-differential expression of *cRLN3* in the pituitary and hypothalamus of chickens. Each data point represents the mean ± SEM of three individual adult chickens (three males or three females) (*N* = 3). **(C)** Effects of the chicken pituitary cell (Pi) extracellular medium (1 × 10^−3^μl to 1 × 10^1^ μl extracellular medium in 30 μl medium, 6 h) on the activation of cRXFP1 expressed in HEK293 cells, monitored by a pGL3-NFAT-RE-luciferase reporter system. HEK293 cells transfected with an empty pcDNA3.1 (+) vector were used as a control and Pi extracellular medium treatment did not change the luciferase activity, indicating the specific action on receptor activation. Each data point represents the mean ± SEM of three replicates (*N* = 3). **(D)** Chicken pituitary single-cell transcriptome data (at https://scrna.avianscu.com/pit/) showing that *cRLN3* is highly expressed in female chicken pituitary gonadotrophs. (d1) Uniform Manifold Approximation and Projection (UMAP) map showing the identified eight pituitary cell clusters. Cells are colored by Seurat clustering and annotated by cell clusters (each point represents a single cell). Gona, gonadotrophs; Lac, lactotrophs; Cort, corticotrophs; Thy, thyrotrophs; FS, folliculo-stellate cells; Endo, endothelial cells; WBC, white blood cells; RBC, red blood cells. (d2) UMAP map showing the expression of *cRLN3* in distinct cell clusters of the female chicken pituitary. The overall expression level of *cRLN3* was high in all pituitary cell clusters of female chickens, and the highest expression level was found in gonadotrophs. (d3) UMAP map showing the expression of *cRLN3* in distinct cell clusters of the male chicken pituitary. In the male chicken pituitary, *cRLN3* was only very poorly expressed in the corticotrophs, lactotrophs, and endothelial cells and was not expressed in other pituitary cell clusters. **(E,F)** Quantitative real-time RT-PCR analysis showing the abundant expression of *cRXFP1* **(E)** and *cRXFP3* **(F)** in various adult chicken tissues. Each data point represents the mean ± SEM of six individual adult chickens (three males and three females) (*N* = 6).

Using qPCR, we examined the mRNA expression of *cRLN3* in adult chicken tissues. As shown in Figure 6A, chicken *RLN3* was abundantly expressed in the pituitary and had a lower abundance in the tissue hypothalamus and ovary. The expression abundance of *cRLN3* mRNA in chicken pituitary can reach a high level with a TPM value (>2000), similar to the expression of the pituitary hormone genes *LHB/FHB/GRP* (gastrin-releasing peptide)/*CART* (cocaine- and amphetamine-regulated transcript) (data not shown). Moreover, *cRLN3* shows a dimorphic expression pattern with high abundance in female chickens. As shown in Figure 6B, the expression abundance of *cRLN3* in the female pituitary was about 60 times higher than that in the male pituitary. In the female hypothalamus, its *cRLN3* expression abundance was about 10 times higher than that in male chickens.

In the present study, the high abundance of *cRLN3* in the chicken pituitary led us to further investigate its distribution in pituitary cell clusters from a single-cell dataset reported by our research group (Zhang et al., 2021). As shown in Figure 6D, *cRLN3* has been found mainly to localize in the gonadotroph cell clusters of female chickens. In addition to the gonadotroph cell clusters, *cRLN3* also was found to localize within the lactotroph cell clusters and the folliculo-stellate cells.

In the present study, the expression of *cRXFP1* and *cRXFP3* genes was also investigated in chicken tissues based on qPCR. *cRXFP1* and *cRXFP3* were detected to be widely but differentially expressed among chicken tissues. As shown in Figure 6E, *cRXFP1* was highly expressed in the infundibulum, uterus, hypothalamus, and the varied functional brain areas (except the cerebellum). In contrast, *cRXFP3* was detected to be predominantly expressed in the hypothalamus and kidney with moderate/weak expression among tissues including the spinal cord, hindbrain, and ovary (Figure 6F).

### The signaling of the secreted cRLN3 from chicken pituitary cells

In the present study, in order to explore whether the chicken pituitary can synthesize and secret the biologically active cRLN3 protein, the extracellular medium of adult female chicken pituitary cells was collected to explore its activation potential for cRXFP1. Using the pGL3-NFAT-RE-luciferase reporter system, we found that cRXFP1 could be activated by the culture medium of pituitary cells with luciferase activity increase in HEK293 cells (Figure 6C).

### The regulation of *cRLN3* mRNA expression in cultured pituitary cells

**FIGURE 7:**
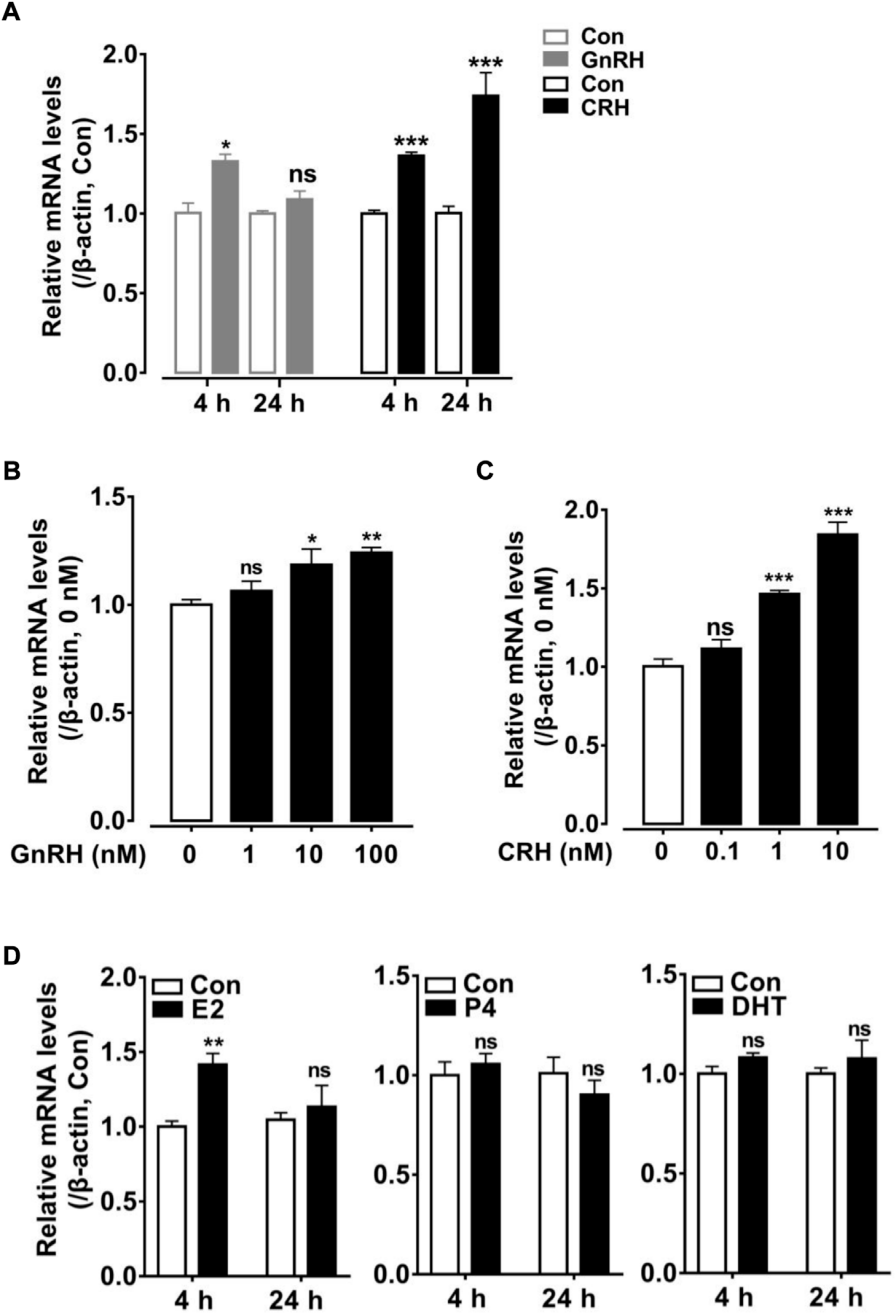
Effects of hypothalamic factors (GnRH or CRH) and sex steroid (E2/P4/DHT) treatment on *cRLN3* expression in cultured chicken pituitary cells detected by qPCR. **(A)** Effects of GnRH (10 nM) or CRH (10 nM) treatment for 4 h, 24 h on *cRLN3* expression in cultured pituitary cells. **(B)** Effects of GnRH (1, 10, and 100 nM) treatment for 4 h on *cRLN3* expression in cultured pituitary cells. **(C)** Effects of CRH (0.1, 1, and 10 nM) treatment for 24 h on *cRLN3* expression in cultured pituitary cells. Each data point represents the mean ± SEM of four replicates (*N* = 4). **(D)** Effects of E2 (100 nM), P4 (100 nM) or DHT (100 nM) treatment for 4 h, 24 h on *cRLN3* expression in cultured pituitary cells. *, *p* < 0.05 vs. control, **, *p* < 0.005 vs. control, and ***, *p* < 0.001 vs. control.

In the present study, the high abundance of *cRLN3* mRNA in the pituitary gland of female chickens led us to further investigate its expression regulation profile. Since *cRLN3* was detected to be localized mainly in the gonadotroph cells, where the GnRHRs and CRHRs were found to be abundantly expressed (Shimizu and Bédécarrats, 2006; Joseph et al., 2009; Zhang et al., 2021), the hypothalamic factors GnRH and CART were employed to investigate their roles in the regulation of *cRLN3* mRNA expression by qPCR. As shown in Figure 7A, the GnRH (10 nM) was able to slightly increase the expression of *cRLN3* mRNA after 4 h of treatment. However, the GnRH (10 nM) was not able to further regulate the expression of *cRLN3* mRNA after 24 h of treatment. In contrast, the CRH was able to significantly increase the expression of *cRLN3* mRNA in a time-dependent manner. In the present study, the different concentrations of the GnRH (1–100 nM, 4 h) and CRH (0.1–10 nM, 24 h) were further employed to examine their effect on the *cRLN3* mRNA expression. As shown in Figures 7B,C, both GnRH and CRH treatments could increase the expression level of *cRLN3* mRNA in a dose-dependent manner, while the stimulation effect of CRH was higher than that of GnRH.

In the present study, the regulatory effects of sex hormones E2 (100 nM), P4 (100 nM), and DHT (100 nM) on the expression of *cRLN3* mRNA in the cultured pituitary cells were also investigated. As shown in Figure 7D, E2 treatment for 4 h could increase the expression level of *cRLN3* mRNA in the cultured pituitary cells. However, the longer E2 treatment (24 h) could not regulate the mRNA expression of *cRLN3*. In the present study, both P4 and DHT treatments could not affect the expression of *cRLN3* mRNA in the cultured pituitary cells.

## Discussion

In the present study, the genes encoding the chicken relaxin 3 peptide (cRLN3) and its two receptors cRXFP1 and cRXFP3 have been cloned for the first time. Functional studies reveal that cRLN3 can activate cRXFP1 and cRXFP3 for downstream signaling. Between the two receptors, cRXFP1 showed a higher binding potency with cRLN3 than cRXFP3, supporting their differential roles among the tissues. In the cultured pituitary cells, the expression of *cRLN3* mRNA is tightly regulated by the hypothalamic factors GnRH and CRH and sex steroid hormone E2. In combination with the detection that the high abundance of *RLN3* mRNA in the pituitary and its co-localization with pituitary hormones genes *LHB/FSHB/GRP*/*CART* in gonadotroph cells in a dimorphic pattern, the present study supports that RLN3 is a novel pituitary hormone involving female reproduction in avian species.

### Chicken *RLN3* gene is the duplicated copy of the ancestral *RLN3* gene

In this study, the chicken *RLN3* gene has been cloned for the first time. Sequence analyses reveal that the precursor protein deduced from cRLN3 shares a low sequence identity with that of other species. However, its A-chain and B-chain from mature proteins still show a higher sequence identity than that of humans, supporting their functional conservation across species (Patil et al., 2017). In addition, the motif RxxxRxxI/V sequences were detected to be conserved in the B-chain, also supporting its function for receptor binding (Bathgate et al., 2013). In the present study, synteny analyses reveal that chicken/duck *RLN3* is orthologous to *RLN3* identified in turtles, frogs, and spotted gars, but not orthologous to *RLN3* identified in humans and mice (Figure 3A). The present study supports that the isolated relaxin 3 gene is likely to be the duplicated copy (*RLN3-L*) from the ancestral relaxin 3 gene, which emerged prior to the divergence of fish (Wilkinson et al., 2005; Yegorov and Good, 2012; Yegorov et al., 2014). Thus, among the lower vertebrates, the gene duplication of *RLN3-L* brings *RLN3a* and *RLN3b* reported in teleost fish (Wilkinson et al., 2005; Yegorov et al., 2014). In chicken, the gene duplication of *RLN3-L* brings the *cRLN3* gene reported in the present study and the predicted *cINSL5* gene (Wilkinson et al., 2005). In contrast, the ancestral *RLN3* gene, which disappeared in chicken, further duplicates in mammals, thus bringing the gene cluster where *RLN1*, *RLN2*, and *RLN3* are located among the mammalian species (Good et al., 2012; Bathgate et al., 2018). In vertebrates, genome duplication and gene gain/loss result in the abundance of relaxin family peptides. The isolation and synteny analyses of *cRLN3* in the present study provide a unique clue for the relaxin family members’ evolution across species.

In the present study, two RLN3 receptors, *RXFP1* and *RXFP3* also have been cloned in chickens. As shown in Figure 2A, chicken RXFP1 shows a high a.a. sequence identity with that of other species. The cloned cRXFP1 shows a large N-terminal, including an LDLa module that is essential for downstream signaling (Halls et al., 2005; Hopkins et al., 2006; Scott et al., 2006) and an LRR region for ligand binding (Scott et al., 2006). In addition, the cloned cRXFP1 also shows the typical seven transmembrane domains with the conserved site for G-protein coupling (Bathgate et al., 2013). Synteny analyses show that the cloned *cRXFP1* is orthologous to *RXFP1* from other vertebrates including humans (Figure 3B), thus suggesting their conserved downstream signaling.

Similar to cRXFP1 to be conserved among the species, the cloned chicken RXFP3 also shares a high sequence identity with that of other species. Like mammalian RXFP3, chicken RXFP3 also contains a short N-terminus, which is crucial for ligand binding and signal transduction (Bathgate et al., 2013). In the present study, the seven transmembrane domains and the conserved site for G-protein coupling also have been detected in chicken RXFP3, supporting it to be the typical G-protein-coupled receptor. Synteny analysis shows that the cloned *cRXFP3* is orthologous to that of other vertebrates including humans, supporting their conserved evolution profile.

### Chicken RLN3 binds cRXFP1 and cRXFP3 for downstream signaling

In the present study, the chicken RLN3-conditioned medium (cRLN3_cm_) could activate cRXFP1 expressed in HEK293 cells, thus stimulating the Gs-cAMP/PKA signaling pathway. Our finding is consistent with the reports in mammals that RXFP1 can be activated by RLN3 (Sudo et al., 2003; Halls et al., 2006). However, only a dose-dependent increase in luciferase activity in HEK293 cells expressing chicken cRXFP1 is detected in the present study which is different from the report in mammalian species that RXFP1 is capable of coupling to Gs and other G-proteins (Gi/o), thus showing a biphasic pattern of cAMP accumulation (Halls et al., 2006; Halls et al., 2007).

In the present study, the chicken RLN3-conditioned medium (cRLN3_cm_) can also activate cRXFP3 through the inhibition of forskolin-stimulated luciferase activity, indicating that cRXFP3 is functionally coupled to the Gi protein (Figure 4A). The present finding is consistent with the findings in mammals (Kocan et al., 2014). In humans, the activation of RXFP3 inhibits forskolin-stimulated cAMP accumulation in a variety of cell lines (Liu et al., 2003b; Liu et al., 2005; van der Westhuizen et al., 2010). The present study supports conserved downstream signaling of RXFP3 between mammalian and avian species.

Although cRLN3 is able to activate both cRXFP1 and cRXFP3, the potency of cRLN3 for cRXFP1 activation is at least 10-fold higher than that for cRXFP3. Similarly, rhRLN3 shows a higher potency for cRXFP1 activation (EC_50_: 2.68 nM) than for cRXFP3 (EC_50_: 41.55 nM). The present study supports that chicken RLN3 is a functional analog of rhRLN3. However, our finding contrasts with that of mammalian species. In mammals, as an endogenous ligand for RXFP3, RLN3 shows a higher potency for RXFP3 activation than for RXFP1 (Liu et al., 2005). The difference may result from ligand–receptor interaction model change during evolution (Bathgate et al., 2003; Bathgate et al., 2018).

In addition to the Gs-cAMP signaling pathway, we also found that cRXFP1 activation can increase intracellular Ca^2+^ levels (Figure 4B). This finding differs from that in mammals that RXFP1 activation cannot increase Ca^2+^ levels (Bani et al., 2002). Like mammalian RXFP3, cRXFP3 cannot activate the Ca^2+^ signaling pathway in the present study, thus supporting its conserved signaling properties between birds and mammals (Liu et al., 2003a; Liu et al., 2003b).

### Chicken RLN3 functions as a novel pituitary hormone involving female reproduction

In the present study, *cRLN3* mRNA expression has been detected to be expressed abundantly in the chicken pituitary (Figure 6). Its abundance in our single-cell transcriptome dataset from the pituitary is very high, reaching a level similar to that of the pituitary genes *LHB/FSHB/GRP/CART*. As the key pituitary hormone in reproduction, LH and FSH are involved in follicular development and ovulation in chickens (Palmer and Bahr, 1992; Oguike et al., 2005). GRP, as a novel “gonadotrophic factor” in chickens (Mo et al., 2017), is reported to be involved in the regulation of reproductive and gastrointestinal activities. CART, expressed abundantly in pituitary cells and regulated by hypothalamic factors CRH and GnRH, is reported to be involved in the regulation of stress and reproductive behavior in domestic chickens (Mo et al., 2015; Mo et al., 2019). Together with the cell signaling experiments which prove that cRLN3 can be secreted from the chicken pituitary cells (Figure 6C), the present study supports that cRLN3 plays its function as a pituitary hormone. Following the detection that its receptors (*cRXFP1* and *cRXFP3*) are differentially expressed in a wide range of chicken tissues, cRLN3 may, on the one hand, be functional in the brain as a neuropeptide (Smith et al., 2011) in mammals (Bathgate et al., 2002; Burazin et al., 2002; Liu et al., 2003b; Smith et al., 2010) and other species including fish (Donizetti et al., 2008). On the other hand, as a pituitary hormone, cRLN3 may also play a role in the endocrine system, targeting wide tissues and, thus, be involved in a wide range of physiological processes.

In the present study, pituitary *cRLN3* mRNA expression was shown to be regulated by the hypothalamic factors GnRH and CRH which further supports cRLN3 to be functional as a pituitary hormone (Figure 7). The present study reveals that CRH potently stimulates the expression of cRLN3 in a dose- and time-dependent manner (Figure 7C). In contrast, GnRH could only weakly stimulate the mRNA expression of *cRLN3* (Figure 7B). Since GnRH is pulsed secreted and negatively regulated by CRH (Ciechanowska et al., 2010), the expression of *RLN3* mRNA may be regulated by CRH. As the key player in the hypothalamus–pituitary–adrenal (HPA) axis of vertebrates, CRH controls multiple physiological processes associated with the HPA axis in birds and mammals (Papadimitriou and Priftis, 2009; Cornett et al., 2013). The RLN3 expression/secretion induced by CRH supports its conserved physiological functions in vertebrates. For example, RLN3 can stimulate rat hypothalamic explants to release GnRH and CRH, and its intraventricular (icv) injection can also increase plasma LH, ACTH, and corticosterone levels (McGowan et al., 2008; McGowan et al., 2014). In chickens, in the transcriptional analyses of newly hatched chicks during fasting and delayed feeding, *RLN3* has been identified in a gene network supporting its important role in nutrition (Higgins et al., 2010).

In the present study, the *cRLN3* mRNA expression was shown to be sexually dimorphic with a much higher abundance in the female pituitary and hypothalamus, strongly suggesting its involvement in female reproduction. Supporting evidence also comes from the high expression of its cognate receptor (*cRXFP1*) in chicken tissues. Similar to rats, *RXFP1* is highly expressed in the uterus involving uterine contractions (Hsu et al., 2002; Vodstrcil et al., 2010). In addition, *cRXFP1* is also detected to be expressed highly in a wide range of female tissues (infundibulum, isthmus, uterus, and vagina), thus supporting its role in reproduction. Supporting evidence also comes from steroid hormone feedback regulation. In the present, E2 has been found to increase the expression of cRLN3 (Figure 7D). As a sex steroid hormone, E2 is mainly secreted by the growing follicles and the ovary, which play an important role in female reproduction, including acting as the growth factor for reproductive organs’ growth (Cooke et al., 1998; Cooke et al., 2021), triggering hypothalamic events *via* a positive feedback system (Micevych et al., 2008; Micevych and Sinchak, 2011), and maintaining follicle growth and maturation (Britt et al., 2004; Drummond, 2006; Padmanabhan et al., 2018). The findings that cRLN3 is involved in female reproductive biology are in line with the report that female teleost fish that RLN3 promotes the production of E2 during follicular development (Wilson et al., 2009; Yang et al., 2020). In another transcriptome analysis between the two divergent chicken breeds, which are in the laying phase and the brooding phase, the expression of *RLN3* mRNA shows downregulation in brooding chickens, thus pointing out its role actively involved in reproductive biology (Shen et al., 2016). The report that RLN3 is actively involved in female reproductive biology also comes from the study by Ghanem and Johnson (2021) in a shotgun proteomics study using isobaric tags to characterize the proteins in chicken ovarian follicles immediately before and after cyclic recruitment. RLN3 is revealed to be distinctively expressed in a total of 1,535 proteins. Based on quantitative PCR, the expression of *RLN3* and its receptors were further revealed to be expressed abundantly in granulosa cells and the theca cells, thus implicating the important role of RLN3 in chicken female reproductive biology.

Till present, a total of five genes (*LHB*, *FSHB*, *GRP*, *CART,* and *RLN3*) have been detected to be abundantly expressed in chicken pituitary gonadotrope cells (Mo et al., 2017; Zhang et al., 2021). Although these genes concentrate on the same cell cluster, these genes vary in sexual distribution patterns, cell co-localization profiles, and the hypothalamus regulation networks. For example, *RLN3* and *GRP* show an abundance in female gonadotrope cells while *LHB*, *FSHB*, and *CART* show a higher expression in male pituitary cells. In addition, all cells expressing *RLN3* overlap with the cells expressing *LHB* and *GRP*. In contrast, the cells expressing *RLN3* only partially co-localize with the cells expressing *FSHB* and *CART* (Zhang et al., 2021). At the hypothalamus regulation level, RLN3 is mainly regulated by CRH while CART is regulated by the GnRH and CRH with equal potency (Cai et al., 2015; Mo et al., 2015; Mo et al., 2019). The present study supports that these genes play differential roles in chicken pituitary function. With their especially complicated regulation networks reported in chickens, the present study enriches our understanding of avian pituitary biology, thus shedding light on their function across species.

In summary, we identified a relaxin peptide (cRLN3) and its two receptors (cRXFP1 and cRXFP3) in chickens. Functional studies have shown that cRLN3 can activate cRXFP1 and cRXFP3, and cRXFP1 is a cognate receptor for cRLN3. Tissue distribution analyses show that *cRLN3* is highly expressed in the female chicken pituitary. Moreover, *cRLN3* in pituitary cells is regulated by hypothalamic factors GnRH and CRH and sex steroid hormone E2. These results suggest that cRLN3 is a novel hormone from the pituitary, which is associated with female reproduction in birds. Undoubtedly, these findings will help us to better understand the structure, ligand–receptor binding, and physiological functions of the relaxin system in birds and other vertebrates.

## Data Availability

Publicly available datasets were analyzed in this study. The scRNA-seq data used in this study have been deposited in the Genome Sequence Archive in the National Genomics Data Center, China National Center for Bioinformation/Beijing Institute of Genomics, Chinese Academy of Sciences, under accession number CRA003604 and are publicly accessible at https://ngdc.cncb.ac.cn/gsa/. A supplementary online web server (https://scrna.avianscu.com/pit/) was developed to facilitate the use of this dataset.
